# Investigating the Outcomes of an Asthma Educational Program and Useful Influence in Public Policy

**DOI:** 10.3389/fpubh.2021.736203

**Published:** 2021-11-26

**Authors:** Hamad Ghaleb Dailah

**Affiliations:** Faculty of Nursing, Jazan University, Jizan, Saudi Arabia

**Keywords:** education, gender, social media, government, public health, public policy

## Abstract

The study was conducted to evaluate the effectiveness of an asthma educational program for asthma control, asthma self-management, asthma knowledge, and patient activation. The study analyzes different demographic variables with the purpose of investigating which asthma patients performed better than others. Based on these demographic characteristics, the study provides several recommendations for various stakeholders. The study is based on a positivist approach since its purpose is to investigate the consequences of an asthma educational program with a view to generalizing the results to a larger population. The study targets public and private hospitals which have applied the asthma educational program in collaboration with the Saudi Initiative for Asthma (SINA). Multiple questionnaires were deployed 263 valid responses were received from patients of public and private hospitals using online and offline data collection method. Several parametric and non-parametric tests were carried out in terms of data analysis. The results reveal that patients in the intervention group obtained high scores and were therefore more knowledgeable and able to control their asthma compared to the control group. Overall, patients in the intervention group performed better in terms of asthma control, asthma self-management and knowledge and awareness. There was a high level of patient activation in this group. In the context of demographic features, it was found that patients who are married and are undergraduate degree holders in employment scored high compared to patients who were young, single, post-graduate degree holders that were mainly self-employed. The results of this study can guide policy makers, SINA authorities, and hospitals as to which demographic category of asthma patients require immediate attention. The significance of asthma educational programmes has increased especially through social media platforms as the number of adult patients continues to increase day by day.

## Introduction

Asthma is recognized as a major non-communicable disease, and there are over 235 million asthma patients across the world (1). The WHO report highlights that ~40 million asthma patients died in 2015 (1). The majority of these patients belong to Arab countries because the health professionals are limited in these nations compared to the number of asthma patients (2–4). Similarly, the numbers of asthma patients are increasing as hospitals are unable to manage them because of limited specialized doctors and staff (3, 5). Approximately 76.7% of asthma patients have expereinced severe asthma attacks in the Kingdom of Saudi Arabia (KSA) (6). Out of 76.7% asthma pateints, 61.6% have visited an emergency department where there are limited numbers of specialized doctors and staff (6). It is found that the percentage of asthma patients has increased from 11.3 to 18.2% in the KSA (7). In this situation, it is important to understand the role and nature of asthma educational programmes, especially in the local and cultural context of KSA. A systematic literature review highlights that the scope of asthma educational programmes. Their numbers have also increased, especially in those countries where there are more asthma patients, more unscheduled patient visits, limited levels of information, and limited numbers of specialized doctors and staff (8–10).

The KSA health care system faces many challenges which have increased the issues for patients with chronic diseases (11, 12). For example, there are lower numbers of allergists, registered respiratory therapists, specialized respiratory nurses, and pharmacists (6, 12, 13). Therefore, the number of emergency and scheduled visits of asthma patients has increased in KSA hospitals (11, 12). A recent study documented that the percentage of asthma patients has also increased (i.e., 11.3–18.2%) in KSA hospitals (7). Recent studies reveal that the number of asthma educational programmes are very limited, and the number of emergency and scheduled visits of asthma patients have also increased in KSA hospitals (7, 14, 15). The KSA government, NGOs, and public hospitals have failed to invest in asthma educational programmes. Therefore, the number of unscheduled visits of asthma patients to hospitals has significantly increased over recent years (7, 11).

The number of allergists, radiologists, pharmacists, and specialized respiratory nurses are very low so patients are unable to make appointments and find it difficult to properly and in a timely manner with health professionals. This has created a critical situation (12, 13, 16). Most KSA hospitals have under-qualified staff, and they are therefore increasingly more dependent on foreign workers who command higher salaries. However, their numbers are also very low, at a time when the number of asthma patients is increasing all over the world (14, 15). It is clear that KSA hospitals and the Ministry of Health must now prepare a digital database to record chronic patient so that they can establish how to manage the sufferers of such diseases. It is now essential that the Saudi health department begins to play a more central role to ensure that all asthma patients can receive more support and lead healthy lives. Community and social support are not very active when it comes to asthma awareness and management. Indeed, SINA is trying to develop awareness, but the majority of patients are not aware of the role and functions of SINA in the context of Asthma control (12, 17).

The existing literature indicates some of the benefits of asthma educational programmes with respect to patient asthma control (18–20). The major aim of this study is to investigate asthma educational programmes as a means to improve the quality of life of sufferers, and to improve medication compliance, asthma knowledge, and patient motivation. These factors can prove helpful in minimizing the numbers of scheduled and unscheduled visits to hospitals. However, many studies of asthma educational programmes have failed to provide relevant information related to what improvements have been achieved with respect to asthma self-management, asthma control, and the quality of life of patients (21–23). Some educational programmes have been unable to bring about any improvements to patients with respect to asthma knowledge (24, 25). Indeed, the actual causes of why patients have limited knowledge even after the delivery of asthma educational programmes are not yet understood (24, 25).

Surprisingly, most existing studies point to high levels of frustration with respect to the intentions and motivations of patients (26–28). Such studies could provide insights about the significance of the age, marital status, gender, and educational level of asthma patients, and the extent to which these influence asthma self-management, patient activation, asthma control, and asthma knowledge (5, 11, 29). Therefore, the present study aims to address this gap by investigating and understanding the influence of demographics descriptors on the outcomes of asthma educational programmes in KSA. Although many studies have explained the role and importance of SINA asthma educational programme in KSA (5, 29, 30), there is no evidence about how these educational programmes influence patients in both intervention and control groups. The present study aims to address that research gap in order to highlight how asthma educational programmes influence asthma self-management, patient activation, asthma control, and asthma knowledge in KSA. The purpose of the study is to enhance the level of understanding about how SINA and other stakeholders in the health sector can improve the health of asthma patients by arranging educational programmes in KSA.

## Literature Review

Most literature in this area focuses on developed countries which have supportive cultures, high levels of asthma awareness and knowledge in social communities, and plenty of specialist staff. Such countries have more asthma educational programmes, and they use technology (31–34). It is unlikely that all cultures, societies, and communities are equal in terms of their asthma knowledge, awareness, healthcare facilities, and in terms of the environmental challenges they face (5, 16, 30). For example, some studies conducted in developing countries indicate there are low numbers of asthma patients, few emergency visits to hospitals, more educational programmes, and limited environmental issues such as hot weather and frequent dust storms which subsequently drive-up rates of allergies (5, 29, 35). Conversely, there are a high number of patients, and limited educational programmes. Patient knowledge about asthma in Arab countries (e.g., Saud Arabia, Oman, Egypt, and Jordan Qatar) is poor (5, 29, 30). And knowledge and awareness about asthma symptoms as well as support from health professionals is very limited in Arab countries (5, 29, 30).

Self-efficacy has been acknowledged through learning theory for over three decades (36). It has long been regarded as a component of social cognitive theory that emerges within behavioral programmes. Enhanced self-efficacy is related to improvements in minimizing asthmatic attacks. It also creates enhanced levels of physical activity, and medication adherence (37). Self-efficacy is considered a key feature of effective symptom management in patients with chronic disease (38). It is highly correlated with the overall functioning of patients who find themselves hospitalized (39). The approach also drives improvement in life standards (40), and improves the 5-year rate of survival amongst patients of chronic disease. Self-efficacy denotes a person's confidence in managing their chronic disease. The key to self-efficacy is confidence so that the patient feels confident enough in dealing with their disease to take medicines on time. Using this approach, they are more likely to make lifestyle changes to better manage the disease (41). Other research has found that higher self-efficacy rates have a positive effect on blood sugar and blood pressure (42). Increased self-efficacy also results in reduced hospitalization rates, enhanced life quality and positive results for health. In contrast, patients who have low levels of self-efficacy fail to maintain their physical exercise levels upon completion of a respiratory rehabilitation program (43). Self-efficacy skills lead to lifestyle improvements and also enable patients to better monitor their illnesses (43, 44).

**Figure 1:**
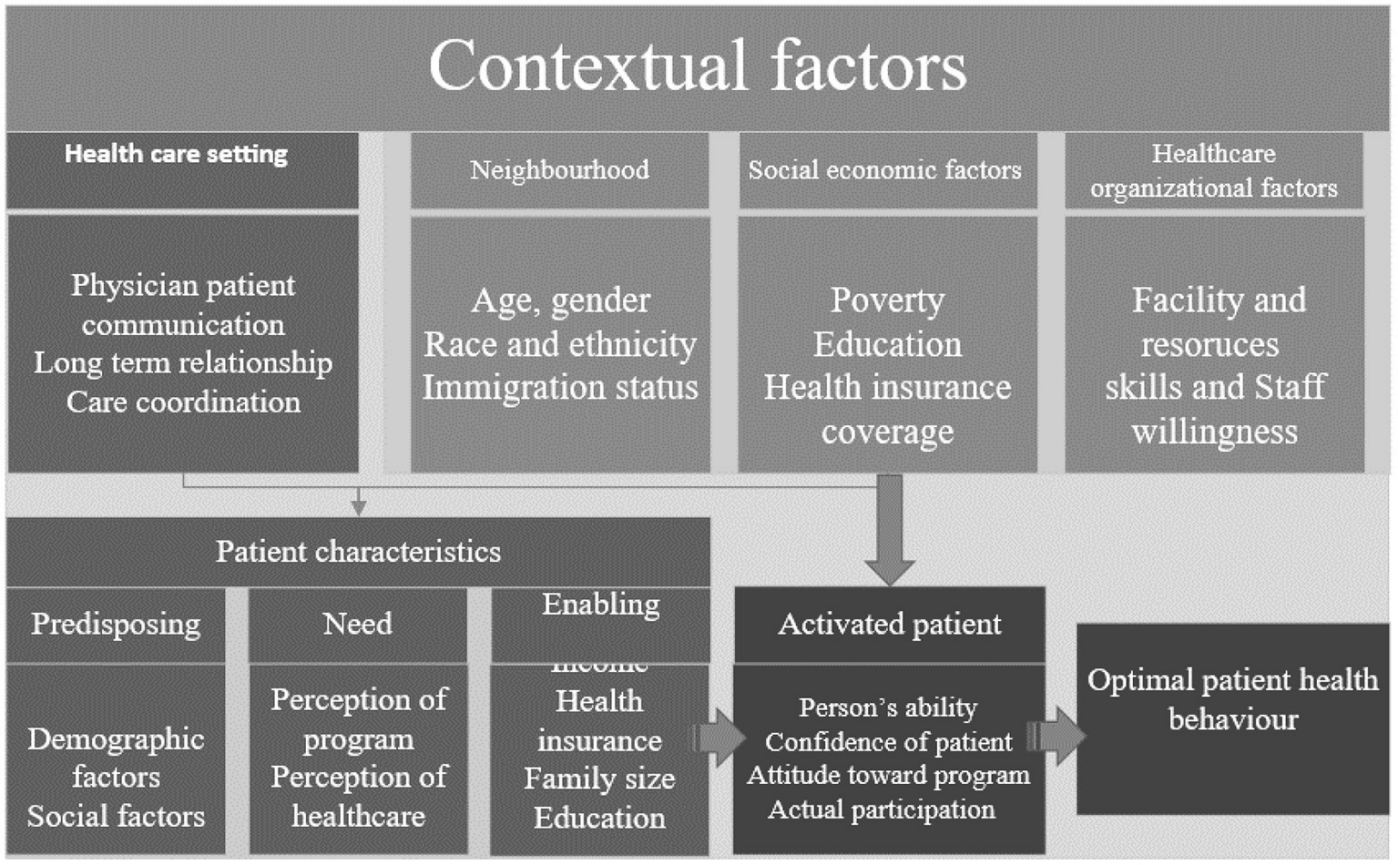
Important contextual factors for Asthma educational programme (Developed by the author).

**Figure 2:**
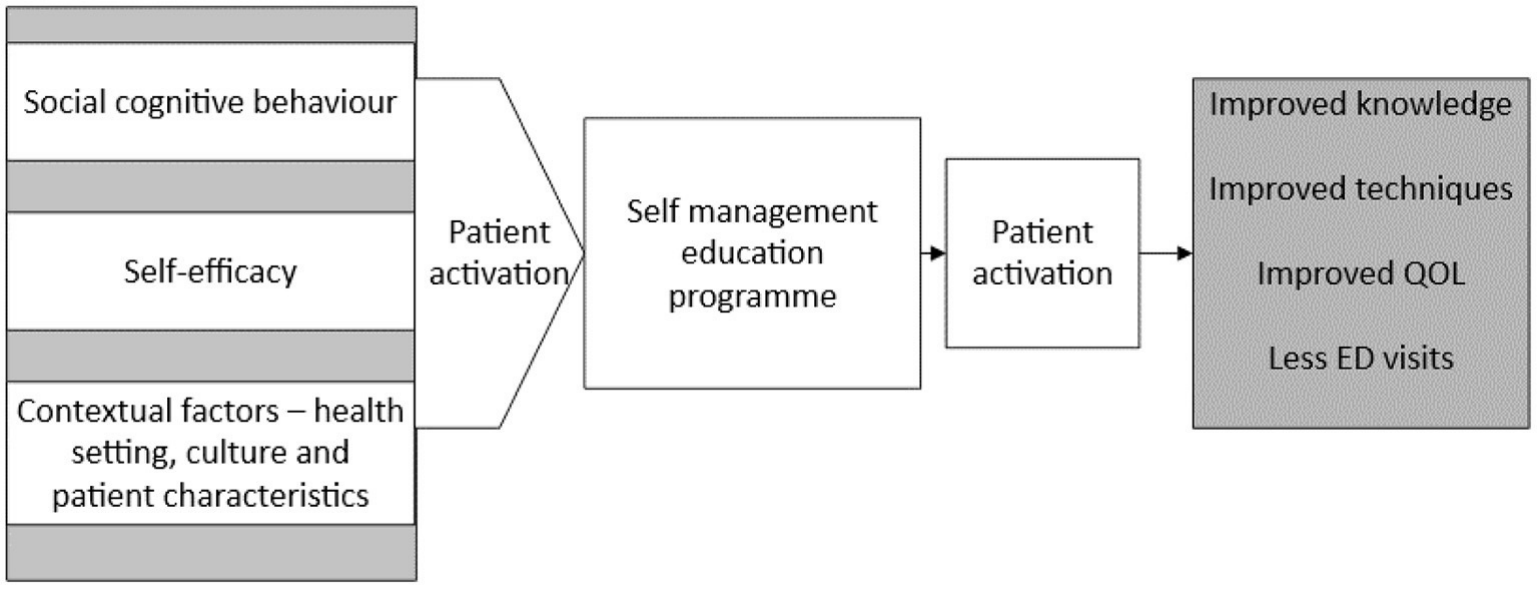
Theoretical framework. QOL, quality of life; ED, emergency department (Developed by the author).

There are some contextual factors that also need to be considered in developing self-management educational programmes for the patient (45). These factors included the patient characteristics and health care setting as shown in Figures 1, 2 below. Similarly, the contextual factors can be considered as the most important attributes of patient activation (46). The environment where patients obtain healthcare is one such contextual factor. There are two kinds of contextual factors: community characteristics and the sites that are the common sources of healthcare (46). Common sources of healthcare, such as hospital emergency departments or surgeries reflect an association between patients and physicians, and this has been shown to strongly influence patient activation.

The extent of literature has highlighted that few KSA hospitals offer high quality health services for asthma patients. Whereas, the majority of hospitals in rural areas receive a high number of asthma patients (11, 12, 47). Furthermore, a number of studies have revealed that KSA rural areas experience high rates of allergies and sandstorms. They have higher populations of smokers, hotter weather and limited sports and exercise opportunities. They consume more dairy items and have low awareness about asthma symptoms and the consequences of asthma (11, 12, 47). A recent study highlighted that the number of adult asthma patients is very high. Therefore, the number of asthma patient deaths have increased across the KSA (17). Similarly, other studies have found that the number of asthma patients are very high compared to health professionals, health facilities, and asthma educational programmes in KSA (13, 15, 16). The existing literature reveals that many patients have attended and have been admitted to KSA hospitals. However, there are limited performance checks and directions for staff (48). Few asthma patients have complained about the poor performance of KSA hospital staff (12, 47). Some asthma patients have found that nurses work very long hours and this impacts on their attention spans, and ability to care for patients (6, 13). This study has been developed based on the following theoretical framework that emerged based on the above literature.

A number of studies have been conducted in KSA, but none of these has identified the extent to which adult asthma patients are motivated to control their asthma without any support (11, 12, 47). It has been found that there are no positive outcomes from asthma educational programmes with respect to reducing emergency visits to hospitals (28, 32, 49). The findings of a recent study revealed that asthma educational programmes decrease the use of inhalers. However, the authors did not reveal how many patients stopped using an inhaler and they did not examine the different techniques they adopted to control their asthma (50). Many studies have examined emergency visits following exposure to education (11, 12, 15). It has been observed that, after the effective delivery of Asthma educational programmes, unscheduled or average emergency patient visits to hospital decrease for all patients (11, 12, 15). It is reported no difference following education, and this study intends to investigate the research questions and hypotheses below (49).

Research Question 1: Is there a significant difference between demographic categories (sex, age, marital status, education, and employment status) in terms of the study variables (Control, Self-Management, Knowledge, and Activation) across program stages?

**Figure 3:**
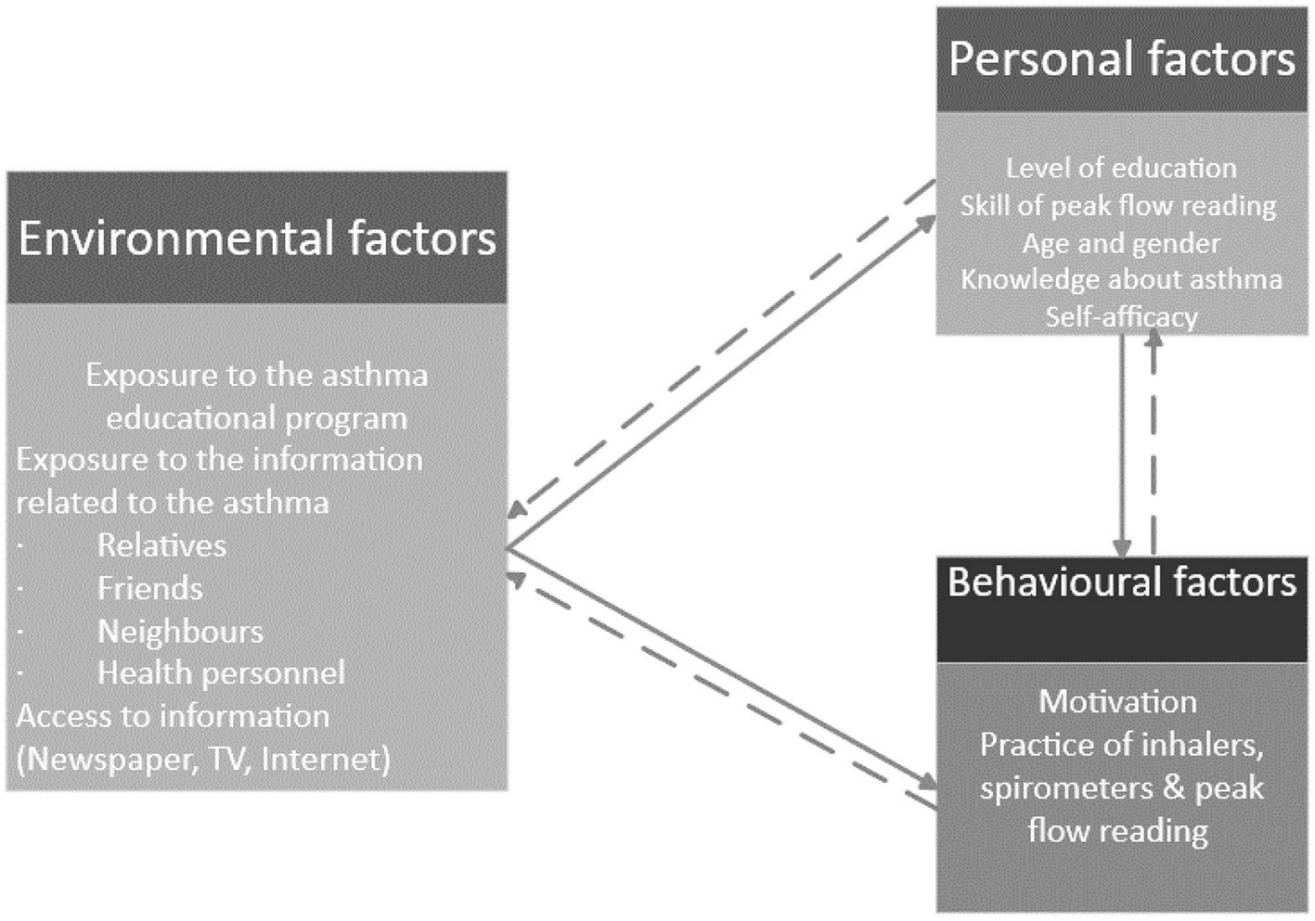
Applies Bandura's social cognitive theory in the context of an asthma education program (Developed by the author).

In order to answer this question (Figure 3), the following hypotheses should be statistically tested.

- *H1a: There is no significant difference between males and females in terms of the study variables (Control, Self-Management, Knowledge, and Activation) across program stages*.
- *H1b: There is no significant difference between age groups in terms of the study variables (Control, Self-Management, Knowledge, and Activation) across program stages*.
- *H1c: There is no significant difference between single and married patients in terms of the study variables (Control, Self-Management, Knowledge, and Activation) across program stages*.
- *H1d: There is no significant difference between education categories in terms of the study variables (Control, Self-Management, Knowledge, and Activation) across program stages*.
- *H1e: There is no significant difference between employment status categories in terms of the study variables (Control, Self-Management, Knowledge, and Activation) across program stages*.

Research Question 2: Is there a significant difference between the three stages of the programme: pre-test, post-test I, and post-test II in terms of the study variables: asthma Control, Self-Management, Knowledge, and Activation?

- *Hypothesis H2a: There is no significant difference between the three stages of the programme: pre-test, post-test I, and post-test II in terms of study variables: asthma Control, Self-Management, Knowledge, and Activation*.

Research Question 3: Is there a significant difference between patient activation levels in terms of the study variables across programme stages?

- *Hypothesis H3a: There is no significant difference between patient activation levels in terms of the study variables across the programme stages?*

## Research Methodology

Understanding the various methodological, epistemological, and ontological dispositions can offer some justification for the selection of a research approach and paradigm. The current study is based on the assumptions of the positivist approach. Based on that approach, the researcher believes that reality is objective, and knowledge may be tested and verified. The outcomes of asthma educational programmes (i.e., patient motivation for asthma self-management, knowledge to control the asthma, and self-determination for patient activation) may vary from patient to patient. It may also vary between developed and developing countries, and between western and Arabic cultures. For example, a number of studies have highlighted that asthma programmes are very limited, and their outcomes in terms of motivation, patient activation, and asthma knowledge are particularly limited, especially in Arab countries (1, 6, 13). Previous, well-known studies have been performed in developed nations where there are high levels of social support, more health professionals and advanced levels of facilities, these nations also have greater awareness and knowledge in social communities about asthma. There is more government support and better planning, and the educational programmes on the self-management of chronic diseases are advanced (9, 51). This study aims to determine the effectiveness of asthma educational programmes with respect to asthma self-management, knowledge of how to control the asthma, and the underlying motivation begin patient activation.

## Population and Sampling

For this study, the researcher has targeted hospitals in which Saudi initiatives for asthma (SINA) have been conducted. These include both public and private sector hospitals such as “Jeddah national hospital, King Abdul-Aziz hospital, Jeddah clinic hospital, Jazan general hospital, and the Saudi German hospital in Jeddah.” The author of this study has served in many hospitals as a health professional, and he therefore has strong social connections which proved helpful in gaining access to people and data. The study used the inclusion and exclusion criteria identified below in terms of selecting respondents (Table 1).

**Table 1:** Inclusion criteria.

**Inclusion criteria**
•Patients diagnosed with any type of asthma with or without co-morbidities.
•Those who are resident in KSA for the duration of the study.
•Aged 18 years or over.
•Able to attend the education program.
•Able to speak and understand English.
**Exclusion criteria**
•Diagnosed but not resident in KSA.
•Those unable to commit to completing the whole of the education program.
•Those who are not able to understand or self-care their asthma.

The researcher has extensive health management work experience from several hospitals in KSA. The social connections of the researcher have been especially useful given that the aim was to collect data about the personal medical histories of asthma patients. Due to these social connections, it was easier for the researcher to select a targeted population for the study. Furthermore, the study uses purposive sampling in order to collect data from patients who were ready to share their personal information and experiences about asthma educational programmes. The present study is cross sectional because it was subject to limited resources such as time, cost and the availability of the researcher and respondents (52, 53). Data were collected from 263 respondents using self-administered questionnaires (see Table 2).

**Table 2:** Descriptive summary of patient activation measurement (PAM).

**Questions**	**Pre-test**				**Post-test I**				**Post-test II**	
	**Control**		**Intervention**		**Control**		**Intervention**		**Intervention**	
	**Mean**	**SD**	**Mean**	**SD**	**Mean**	**SD**	**Mean**	**SD**	**Mean**	**SD**
PAM total mean and SD	2.81	0.303	2.75	0.417	2.82	0.338	3.16	0.377	3.62	0.419

*SD, standard deviation*.

## Ethical Considerations

Ethical approval was obtained from the Saudi Ministry of Health (MOH) prior to the study commencing. It is mandatory for any person who wishes to perform research, or who requires to collect personal data from any hospital in the KSA to obtain ethical approval from the ethics committee of the MOH. Therefore, before starting the research, an application was presented to the MOH, consisting of a short summary of the target sample and region. The MOH committee accepted the application for ethical approval. It was further explained to the participants that, once they agreed to participate in the study, they were equally free to withdraw their participation at any time, without any recourse.

## Data Collection and Analysis Technique

Multiple data collection instruments were adopted to fulfil the proposed objectives of this study. For example, an asthma control instrument was adopted from a previous study (54) and used in this study. This tool consists of a five-point Likert scale. As such, a number of asthma self-management, asthma knowledge and patient activation instruments were adopted from previous studies (55, 56) and used in this study based on a five-point Likert scale. These four data collection instruments (i.e., asthma control test, asthma self-management questionnaire, and the asthma knowledge) have been individually used in many previous studies in different countries, and they have been found to offer high reliability scores (54, 57). Therefore, the present study uses all of these data collection instruments with the purpose of determining appropriate asthma educational programmes for Saudi patients. These instruments have been checked though a pilot study and found to offer high reliability scores (i.e., asthma control test: 0.78; asthma self-management: 0.72; asthma knowledge: 0.81, and patient activation: 0.69). These data collection instruments were subsequently used to carry out further statistical tests. *T*-test and analyzes of variance (ANOVA) were undertaken where data were normally distributed. Kruskal–Wallis and *Post-hoc* multiple comparisons tests tests were performed where the research data were not normally distributed. Table 3 shows the changes within the intervention groups across different programme stages based on the Kruskal–Wallis test.

**Table 3:** Change within intervention group across different program stages.

**Study variables**	**Descriptive outcomes**				**ANOVA statistics**			
	**Pre-test**		**Post-test I**		**Post-test II**		
	**Mean**	**SD**	**Mean**	**SD**	**Mean**	**SD**	**F**	**Sig**.
Control	15.37	3.650	18.10	3.524	20.19	3.478	28.750	<0.000
Self-management	22.78	13.007	58.57	18.775	82.86	16.897	210.476	<0.000
Knowledge	8.39	2.944	12.10	2.838	14.40	2.426	75.795	<0.000
Patient activation	2.75	0.417	3.16	0.377	3.62	0.419	72.176	<0.000

## Saudi Initiative for Asthma Educational Programme

Studies have shown that patients feel more comfortable learning about asthma in their local language, and when interacting with experts. Such experts tend to use words and gestures that are easy to understand, and which can enhance knowledge. Age, gender, education, ethnicity, patient perception, patient behavior, patient motivation, and local culture are some important factors which can influence patient activation and patient control over asthma (58, 59). One of the landmark developments for asthma in KSA was SINA. This was introduced by the Saudi Thoracic Society (STS) in 2009. The government of KSA implemented various new SINA strategies to improve asthmatic patients' health status. These provide up-to-date guidelines for healthcare workers on how best to manage patients with asthma (60). These guidelines provide a clear pathway for the diagnosis and management of asthma, focusing on the need for education for both nurses and patients in Saudi Arabia (61). SINA was originally developed in 2008 by Saudi experts with long outstanding experience and honorable academic background in their field. While developing SINA, these experts used local literature and the latest evidence and knowledge about current KSA settings (62). SINA placed greater emphasis on understanding clinical presentation, epidemiology, pathophysiology, and medications.

The SINA consulted the UK and other international materials before designing the Saudi asthma educational programme. This asthma educational content was designed in the native language (Arabic) so that it could increase Saudi patients' understanding, motivation, activation, and awareness. The SINA asthma educational content includes written education materials, basic definitions of asthma, disease management and control protocols, medication pictures and descriptions, and demonstrations about the use of inhalers and the role of peak flow. It includes symptom diaries, advice on environmental control and trigger, dietary counseling, weight management advice, smoking cessation guidance, further guidance of the management of chronic conditions such as heart disease and hypertension, and medication compliance information. Across a two-day session, SINA delivers asthma educational programmes using skype video sessions, Microsoft teams, social media, face to face lectures, and brochures. The video lectures are shared on social media accounts of different hospitals so that it can be accessible for people across the Saudi Arabia as it can enhance the public engagement and knowledge.

## Findings and Analysis

Simple random sampling was used to select respondents for intervention and control groups. Based on random sampling, 129 respondents were selected for the intervention group while 134 respondents were selected for the control groups (selected from offline and online groups equally). These respondents were consistent across all stages of the pre-test, post-test I, and post-test II. Approximately 450 questionaries were distributed (offline and online respondents) to the targeted patients across four hospitals in the KSA. Some 330 questionnaires were returned, and some had high rates of missing values. Out of these 330 questionnaires, 263 were deemed valid and acceptable for further analysis. The majority of respondents were male in both the intervention and control groups. The highest number of patients (i.e., 44 and 34 patients) with respect to age categories (i.e., 18–25 years and 51–65 years). Most of the patients were married and had completed high school and post graduate degrees (see Table 4).

**Table 4:** Patients' demographic features (*N* = 263).

	**Control (*n* = 134)**	**Intervention (*n* = 129)**	**Total (*n* in%)**
**Gender**			
Female	63	59	0.46
Male	71	70	0.54
**Age group**			
18–25	44	33	0.29
26–35	33	29	0.23
36–50	29	33	0.24
51–65	28	34	0.23
**Marital status**			
Single	45	59	0.40
Married	89	70	0.60
**Education**			
High-school or less	49	35	0.31
Undergraduate level	43	45	0.33
Postgraduate level	42	49	0.34
**Employment status**			
Employed	44	33	0.29
Unemployed	18	24	0.15
Self-employed	27	21	0.18
Retired	22	28	0.19
Looking for work	23	23	0.18

Table 5 has highlighted that no significant improvement was found with respect to asthma control over the three different stages. That is to say that the pre-program stage showed that results were achieved before the asthma education program. Post-program stage-I yielded results after 3 months of the asthma program, and post-program II highlighted outcomes after 6 months of training. A very positive change was noticed in the intervention group of patients across the three stages. Table 5 illustrates the difference in patient scores in individual items across the different study stages (see Table 5).

**Table 5:** Asthma Control Test (ACT) descriptive values.

		**Responses scale**						
**Program stage**	**Patient group**	**1**	**2**	**3**	**4**	**5**	**Statistics**	
**(1). In the past 4 weeks, how much of the time did your asthma keep you from getting as much done a t work, school or at home?**								
		**All of the time**	**Most of the time**	**Some of the time**	**A little of the time**	**None of the time**	**Mean**	**SD**
Pre-test	Control	16 (25.4)	7 (11.1)	16 (25.4)	17 (27.0)	7 (11.1)	2.87	1.362
	Intervention	16 (25.8)	18 (29.0)	11 (17.7)	13 (21.0)	4 (6.5)	2.53	1.264
Post-test I	Control	12 (19.0)	12 (19.0)	14 (22.2)	16 (25.4)	9 (14.3)	2.97	1.344
	Intervention	17 (27.0)	18 (29.0)	19 (30.6)	15 (24.2)	10 (16.1)	3.27	1.058
Post-test II	Intervention	1 (1.6)	6 (9.7)	20 (32.3)	20 (32.3)	15 (24.2)	3.68	1.004
**(2). During the past 4 weeks, how often have you had shortness of breath?**								
		**More than once a day**	**Once a day**	**3–6 times a week**	**Once or twice a week**	**Not at all**	**Mean**	**SD**
Pre-test	Control	4 (6.3)	6 (9.5)	17 (27.0)	28 (44.4)	8 (12.7)	3.48	1.045
	Intervention	9 (14.5)	7 (11.3)	20 (32.3)	22 (35.5)	4 (6.5)	3.08	1.149
Post-test I	Control	5 (7.9)	6 (9.5)	25 (39.7)	20 (31.7)	7 (11.1)	3.29	1.054
	Intervention	1 (1.6)	9 (14.5)	10 (16.1)	29 (46.8)	13 (21.0)	3.71	1.014
Post-test II	Intervention	1 (1.6)	4 (6.5)	11 (17.7)	20 (32.3)	26 (41.9)	4.06	1.006
**(3). During the past 4 weeks, how often did your asthma symptoms (wheezing, coughing, shortness of breath, and chest tightness or pain) wake you up at night or earlier than usual in the morning?**								
		**4 or more nights a week**	**2 or 3 nights a week**	**Once a week**	**Once or twice**	**Not at all**	**Mean**	**SD**
Pre-test	Control	5 (7.9)	10 (15.9)	16 (25.4)	17 (27.0)	15 (23.8)	3.43	1.241
	Intervention	3 (4.9)	13 (21.3)	16 (26.2)	21 (34.4)	8 (13.1)	3.30	1.101
Post-test I	Control	3 (4.8)	16 (25.4)	14 (22.2)	16 (25.4)	14 (22.2)	3.35	1.220
	Intervention	2 (3.2)	5 (8.1)	18 (29.0)	23 (37.1)	14 (22.6)	3.68	1.021
Post-test II	Intervention	-	1 (1.6)	13 (21.0)	21 (33.9)	27 (43.5)	4.19	0.827
**(4). During the past 4 weeks, how often have you rescue inhaler or nebulizer (such as albuterol)?**								
		**3 or more times per day**	**1 or 2 times per day**	**2 or 3 times per week**	**Once a week or less**	**Not at all**	**Mean**	**SD**
Pre-test	Control	4 (6.3)	17 (27.0)	20 (31.7)	6 (9.5)	16 (25.4)	3.21	1.272
	Intervention	7 (11.7)	7 (11.7)	17 (28.3)	15 (25.0)	14 (23.3)	3.37	1.288
Post-test I	Control	8 (12.7)	18 (28.6)	12 (19.0)	5 (7.9)	20 (31.7)	3.17	1.465
	Intervention	1 (1.6)	7 (11.3)	17 (27.4)	21 (33.9)	16 (25.8)	3.71	1.030
Post-test II	Intervention	2 (3.2)	2 (3.2)	12 (19.4)	23 (37.1)	23 (37.1)	4.02	1.000
		**Not controlled at all**	**Poorly controlled**	**Somewhat controlled**	**Well controlled**	**Completely controlled**	**Mean**	**SD**
**(5). How would you rate your asthma control during the past 4 weeks?**								
Pre-test	Control	3 (4.8)	7 (11.1)	24 (38.1)	14 (22.2)	15 (23.8)	3.49	1.120
	Intervention	3 (4.8)	14 (22.6)	20 (32.3)	14 (22.6)	11 (17.7)	3.26	1.144
Post-test I	Control	4 (6.3)	15 (23.8)	24 (38.1)	11 (17.5)	9 (14.3)	3.10	1.118
	Intervention	–	4 (6.5)	20 (32.3)	27 (43.5)	11 (17.7)	3.73	0.833
Post-test II	Intervention	–	1 (1.6)	11 (17.7)	22 (35.5)	28 (45.2)	4.24	0.803

The Asthma Self-management Questionnaire (PSMQ) included 16 questions that patients were shown. They were asked to choose a letter that corresponded to their answers. Asthma self-management was measured by counting the number of correct responses made by patients to 16 questions. It was found that patients achieved very low scores across the stages in the control group. However, patients in the intervention group performed very well, and showed that they can self-manage their asthma (see Table 6).

**Table 6:** Asthma Self-management Questionnaire (ASMQ) summary of correct and incorrect responses.

**Question**	**Pre-test**						**Post-test I**						**Post-test II**		
	**Control**			**Intervention**			**Control**			**Intervention**			**Intervention**		
	**✗**	**✓**	**DK**	**✗**	**✓**	**DK**	**✗**	**✓**	**DK**	**✗**	**✓**	**DK**	**✗**	**✓**	**DK**
Average results of all the questions of ASMQ	38 (60.1)	15 (21.5)	19 (3.7)	38 (61.3)	7 (11.3)	17 (27.4)	44 (69.8)	3 (4.8)	16 (25.4)	19 (30.6)	43 (69.4)	0 (0.0)	6 (9.7)	56 (90.3)	0 (0.0)

*✓= Correct Responses, ✗ = Incorrect Responses, DK = I don't Know*.

The Asthma Knowledge Questionnaire (AKQ) which is based on 17 questions was used in this study. This questionnaire is based on various answer options, such as the number of correct, incorrect, and unknown answers. In Table 7, the number of incorrect, correct, and unknown answers are reported in terms of frequencies and percentages for each patient group across the various program stages. It was found that the number of correct responses amongst control group patients were the same in different programme stages, while variation was seen in the intervention group. That group saw increases in post-test I and post-test II stages (see Table 7).

**Table 7:** Asthma Knowledge Questionnaire (AKQ) summary of correct and incorrect responses.

**Questions**	**Pre-test**						**Post-test I**						**Post-test II**		
	**Control**			**Intervention**			**Control**			**Intervention**			**Intervention**		
	**✗**	**✓**	**DK**	**✗**	**✓**	**DK**	**✗**	**✓**	**DK**	**✗**	**✓**	**DK**	**✗**	**✓**	**DK**
Average results of all the questions of AKQ	5 (8.9)	49 (66.8)	9 (13.3)	9 (12.5)	45 (72.6)	8 (11.9)	6 (9.5)	53 (84.1)	4 (7.3)	4 (6.5)	58 (93.5)	0 (0.0)	2 (3.2)	60 (96.8)	0 (0.0)

*✓= Correct Responses, ✗ = Incorrect Responses, DK = I don't Know*.

Patient activation was measured using 10 questions that were answered using a 5-point Likert scale where zero means “Not Applicable,” 1 means “Strongly Disagree,” 2 means “Disagree,” 3 means “Neutral,” 4 means “Agree,” and 5 means “Strongly Agree.” Means and standard deviations for individual items were calculated for the 10 questions. The mean scores of the patients in the control groups improved across different programme stages. However, the improvement rate of the control group was lower as compared to the intervention group across different asthma programme stages (see Table 7 descriptive summary of PAM).

The results presented in Table 3 show a change within the intervention group across the different programme stages. Patients experienced improved asthma control, asthma self-management, and asthma knowledge at the pre-test, post-test I, and post-test II stages. However, clear and strong changes were only observed in patient asthma self-management at all three stages. The patient activation mean values suggests only a very minor impact from the asthma educational programme with respect to patient activation across all stages (see Table 3).

## Discussion

The primary objective of self-management educational programmes is to enable patients to effectively manage and control their health conditions, particularly in the case of asthma by using various programme elements. It was found that self-management educational programmes can improve outcomes for asthma patients (28, 31). They can also increase self-efficacy (63) and create positive self-management behavioral changes like inhaler use, medication adherence, regular exercise, and follow-up visits (63, 64). They can drive understanding and knowledge (64, 65), and bring about reductions in both the inappropriate usage of medications and unscheduled visits (31). It was also observed that such self-management programmes facilitate the identification of hurdles to self-management, and they can inform strategies that can improve understanding and health literacy (28). However, no previous study of self-management has examined all these elements. In addition, there has been no focussed study of the Saudi Arabian context, and so the unique contribution of this research is clear. Individuals with asthma can take better care of themselves if they regularly visit their doctors and attend asthma self-management education programmes. Patients should be able to resolve this issue for themselves and also have better self-encouragement in this regard. However, individuals with asthma who have insufficient financial resources, narrow social networks, little access to advanced technology (66) and less accessibility to specialized staff and hospitals are not very aware of the negative consequences and side effects of asthma (67).

To meet the objectives of this study, asthma patients that were willing to attend educational sessions on asthma control and share their experiences related to such programmes were targeted. The researcher conducted normality tests and found that the data were normal based on statistical procedures. Using quantitative methods, the present study tested patient knowledge and awareness using three different stages (i.e., the pre-program stage before attending an asthma educational programme; the post-program stage-I, after 3 months of an asthma training programme; and post-program II after 6 months of training). Furthermore, the study looked at two groups (i.e., the intervention and control group) of asthma patients. Four different instruments were applied, i.e., the Asthma Control Test (ACT), Asthma Self-management (ASMQ), Asthma Knowledge (AKQ), and Patient Activation Measurement (PAM). First, we analyzed the improvements and experiences of patients—before and after educational programme—who were selected for inclusion in a control and intervention group. For this purpose, the study used the Asthma Control Test (ACT) and found no significant positive change in the control group over the three program stages. The mean scores increased for patients in the intervention group over the three program stages.

Second, the study employed the Asthma Self-management (ASMQ) questionnaire with the purpose of obtaining information related to flare-ups, the use of inhalers, the use of medicines and awareness about symptoms and action planning. This questionnaire also took account of exercise, and any other help received. The number of correct responses was higher for the intervention group compared to the control group. The results highlight that patients in the intervention group scored more, and were thus able to manage their asthma effectively as compared to patients in the control group. Third, the current study used the Asthma Knowledge questionnaire (AKQ) with the purpose of obtaining information related to disease (i.e., inflammatory, contagious, hereditary), awareness, the use of drugs, the negative consequences of asthma, awareness about possible treatments, the use of an inhaler and other medications, self-motivation, and participation in health activities. It was found that participants in the intervention group obtained higher scores and were thus seen as more knowledgeable as compared to patients in the control group. Despite this, the majority of patients suffering with asthma in Saudi Arabia were found to lack the necessary knowledge that is required to control the disease and enable them to achieve a good quality standard of living (13). Therefore, this study has examined how patient activation can play a role, especially in the presence of the Covid-19 outbreak when health resources have become limited across the globe.

The adopted and used a Patient Activation Measurement (PAM) questionnaire with the objective of obtaining data about self-efficacy, self-motivation, self-determination, self-confidence, and a willingness to attend a medical follow-up, and follow an eating schedule. The statistical value of mean is higher in those patients who were members of the intervention group. In addition, participants in the intervention group obtained higher scores and were thus seen as having more self-efficacy, self-motivation, self-determination, and self-confidence. They were more willing to attend a medical follow-up and follow a well-planed eating schedule compared to patients in the control group. It has been known for some time that the occurrence of asthmatic attacks increases when health professionals are unable to provide the appropriate health education (68, 69). Therefore, asthma education can increase patient activation.

Several statistical tests were used with the purpose of establishing the influence of demographic features (i.e., age, gender, employment, education, and marital status) on asthma control, self-management for asthma, knowledge and awareness, and patient activation. The Mann–Whitney test was used with the purpose of measuring the differences between males and females. The findings suggest that males showed a good intention to control asthma at post-program I. Females obtained a higher score and were more active in controlling their asthma at post-program II stage. Meanwhile, a 2019 study of 1,009 patients found 30.1% suffered with asthma, with higher asthma control reported by males and those with higher education (13). It was found that males have achieved high scores for self-efficacy, self-motivation, self-determination, self-confidence, medical follow-up, and following a planned eating schedule. However, there was no difference in terms of the scores in the context of asthma self-management, knowledge, and awareness.

Existing studies highlight that the intensity of asthma varies due to the environment, and ages of people that vary across countries (70, 71). This study investigated the significance of age with respect to asthma self-management in the context of Saudi Arabia. The results reveal that patients who fall into the age category of 18–25 years performed very poorly with respect to controlling their asthma. Patients who fall into the age categories of 26–35 years and 36–50 years performed effectively in terms of controlling their asthma compared with younger patients (i.e., 18–25 years). Third, the present study performed a Mann–Whitney test with the purpose of measuring the differences between unmarried patients and married patients. It found that married patients were more knowledgeable and had a greater intention to control their asthma as compared to those who are unmarried. However, there was no difference found between married and unmarried patients with respect to patient activation and the self-management of asthma.

Fourth, the researcher used *post-hoc* multiple comparison tests with the purpose of measuring differences in the context of education (i.e., high school, undergraduate level, and post-graduate level). During the pre-test stage, those patients who had undergraduate degrees scored higher in terms of their ability to control their asthma as compared to those patients who had high school certificates and post-graduate degrees. Furthermore, patients who had an undergraduate education as part of the control group performed very effectively. In terms of the post-program-I stage, patients who had an undergraduate education scored very highly with respect to controlling their asthma and in terms of their self-management, and patient activation. Those patients who have undergraduate level degree had better knowledge than patients with high school. Fifth, Kruskal–Wallis and *post-hoc* multiple comparisons tests were carried out with respect to establishing the impact of employment status (i.e., organizational employee and self-employed) on asthma control, self-management, knowledge and awareness, and patient activation across the various stages. In the context of post-program-I and post-program II, the findings reveal that employed patients scored higher and were more knowledgeable compared to those patients who were self-employed. There were no significant differences found with respect to employment status and its impact on other variables (i.e., asthma control self-management, and patient activation).

The self-management concept develops when one becomes aware about the significance of self-care amongst sufferers of chronic disease. The healthcare providers in this regard must share information regarding chronic disease with patients (72, 73). Factors which influence self-management involve self-care at the individual cognitive level, and include psychological, physical, cultural, and social factors (4). The concept of self-management is aligned with that of self-care and involves the associated concepts of the self-monitoring of disease, and symptom management. These concepts are then mediated by self-efficacy. Moreover, Albert Bandura introduced the term self-management. Generally, the significance of self-management is that it prepares people with chronic diseases to manage their healthcare plans more actively.

Therefore, hired nurses and professionals of asthma educational programmes have a certain understanding about religious beliefs. They might therefore believe in the efficacy of educational programmes. Some quote references from the Quran, where Allah (God) recommends learning until death and the centrality of medication adherence. The Quran also speaks of the value of prayers, of care, and of diets. Shared knowledge from the Quran also increases the motivation and confidence of Saudi asthma patients. As a result, asthma patients in the intervention groups performed much better in terms of asthma control, self-management, knowledge and awareness, and patient activation.

Some interesting results were observed during the quantitative analysis from this study. First, males obtained very low scores on the Asthma Control Questionnaire (ACQ) compared to females at the pre-program stage. However, males scored very high in terms of asthma control at post-program II. This suggests that males yield more benefits from this educational program, and they are more interested in controlling their asthma compared to females. Second, young patients (18–25 years) performed very poorly with respect to controlling their asthma and in terms of their desire to receive more knowledge about asthma. Therefore, more training programmes that could enhance the level of awareness, knowledge, motivation, and self-efficacy amongst patients seeking to control asthma would be useful for young patients in the KSA.

Patients with asthma who are not diagnosed and managed appropriately can cause a substantial burden to individuals and their families. More than 80% of asthma deaths occur due to poor knowledge amongst low and lower-middle income countries (1). There is limited understanding about married and single asthma patients with respect to asthma control. The results of this study highlight that married patients are more willing to control asthma and more likely to seek knowledge about the illness during the various stages of this educational programme. One of the reasons for this is that married patients may assume more responsibility with respect to managing their health. Single patients performed poorly in terms of controlling their asthma and were less inclined to seek out knowledge of asthma. Therefore, it is crucial to arrange motivational sessions for schools, colleges, and universities so that unmarried patients can enhance their level of their knowledge and motivation to overcome chronic diseases. Fourth, patients who have received high school educations and post-graduate level degrees performed poorly with respect to asthma control, self-management, and patient activation.

The findings show that there is no proper education and awareness about asthma amongst patients who obtained higher education degrees in universities in the KSA. Therefore, the health ministry and education department must work together to control and manage these chronic diseases at the initial stages. Fifth, patients working in organizations performed much better with respect to asthma control, self-management, knowledge and awareness, and patient activation. One of the reasons for this is that they are more knowledgeable, and active, and more likely to take advice from their colleagues and social communities. Patients who have their own businesses performed very poorly with respect to asthma control, self-management, knowledge and awareness, and patient activation. Therefore, there needs to be more of a focus on individuals who are self-employed, but who have limited knowledge, time, and intention to learn about how to self-manage asthma.

Overall, it can be concluded that levels of asthma were uncontrolled before the delivery of asthma educational programmes, but patient knowledge and health conditions improved after stage I and stage II. Females did not receive the maximum benefits from the educational programme as compared to males. Previous studies have explained the importance of family in asthma control but did not reveal the main influencers of family that can help to control asthma (74, 75). Therefore, it is important to establish how the level of engagement and activation can increase amongst females. It is also useful to engage female patient families (i.e., husband and mother) so that they can create a family influence and a high level of care which is very useful to overcome the disease.

There is, however, a significant difference in the performance of the control group with respect to seeking knowledge about asthma. In the intervention group, patients performed much better in terms of asthma control, self-management, knowledge and awareness, and patient activation. In the intervention group, most patients had a limited level of information about asthma self-management, and little knowledge about treatments at the pre-stage. However, their knowledge significantly improved during the later stages. Finally, this educational program improved skills amongst patients who were more active and motivated to control asthma.

In summary, findings reveal that the asthma educational program has successfully increased levels of knowledge and awareness amongst targeted patients. The present study was based on two groups: the control and intervention group. The aim was to analyse the effect of an educational programme where the aims is to increase controls over asthma, patient activation, and patient knowledge. The results reveal that no improvement occurs with respect to controlling asthma, patient activation, and patient knowledge amongst patients who were included and tested in the control group. Exceptional improvements occurred with respect to patients' control over asthma and their activation, and knowledge. Both stages (i.e., stage I and stage II) showed improvements in asthma control, patient self-management, asthma knowledge, and compliance with medication. This led to reduced visits to emergency departments. In the context of demographic features, the present study found different scores with respect to age, gender, employment, education, and marital status. For instance, it was found that scores for female patients were excellent in the pre-test stage, while male patients scored higher after the intervention stage as compared to females.

## Conclusion

Overall, it was found that members of the intervention group performed better in the context of asthma control and asthma self-management. They had more knowledge and awareness of asthma, and higher levels of patient activation. In addition, the study used a one-way ANOVA test with the purpose of monitoring improvements in patients in the intervention groups during the programme stages. The results show that the mean scores of the intervention group were higher at the post-programme II stage, as compared to the pre-programme and post-programme stages. Therefore, it can be concluded that the intervention group members have more control over their asthma. They are more able to manage their asthma. They have greater knowledge and awareness, and higher patient activation during the post-programme II stage. From the perspective of age differences and the influence of thee on the intervention group, patients who fall within the range of 26–35 and 36–50 years of age performed well in the pre-programme, post-programme I, and post-programme II stages. The differences between scores indicate that males show a higher interest in asthma educational programmes and therefore achieved higher scores at the end of program. Moreover, married patients show more interest in increasing their awareness and control over asthma. They demonstrate better self-management, asthma knowledge, and consistency in the use of medication. Single patients (i.e., unmarried) demonstrated very low levels of interest with respect to improving their health.

The study finds that the age is a significant factor in the control of asthma. For example, older patients scored lower in terms of controlling the level of their asthma as compared to patients who were younger and motivated to learn and control the illness. Those respondents who had higher levels of education and were more aware about the symptoms of asthma showed more interest in receiving the maximum level of knowledge and confidence in the context of resolving asthma. The findings reveal that patients who attended asthma education programme were able to create a proper *self-management plan* against the disease. These respondents are continuously interacting with family doctors and attending asthma self-management educational programmes. Therefore, these respondents are more motivated to overcome shortness of breath, wheezing, coughing, and chest tightness. The findings reveal that self-management education programmes have increased skills amongst patients who now understand how they can minimize the intensity of their asthma. From an educational perspective, patients who have undergraduate level degrees scored higher when it came to controlling their asthma compared to patients who had a high school education. Conversely, patients who had postgraduate degrees performed poorly compared to patients who had undergraduate level degrees. From an employment perspective, patients who had higher designations showed more interest in increasing their awareness and knowledge about asthma control. Patients showed little interest in terms of enhancing their education and control over asthma compared to employees who had higher designations. In terms of the programme stages, the intervention stage had a significant and positive impact on levels of awareness amongst asthma patients.

### Implications

✓ From a practical point of view, young patients (i.e., 18–25 years age) have very poor knowledge with respect to asthma control at the post programme stages. The government, the ministry of health and health professionals must take immediate action to engage and enhance young patient activation through social media platforms where they are spending most of their time.
✓ The findings reveal that those patients who have a post-graduate degree demonstrated very poor knowledge with respect to asthma control. These findings show that colleges and universities are not involved in educating people with respect to symptoms, consequences and the risks of asthma. The KSA government, the ministry of health and SINA should design online educational programmes for colleges and universities that can easily monitor and engage maximum targeted population.
✓ The results of the post programme stages showed that, compared to females, males could not reap the same benefits from asthma educational programmes. It is important therefore to improve patient activation and motivation by involving family members (i.e., the wife, mother, father, and children).
✓ The outcomes of this study indicate that mature patients who are married, as well as those who are employed, have more social interactions and awareness about asthma. However, unmarried and self-employed patients demonstrate poor knowledge with respect to asthma control and management. Therefore, it is important to target young as well as self-employed patients to minimize asthma problems in young adult patients.
✓ The results indicate that no proper mechanism has been developed with respect to asthma educational programme experts and staff. SINA and the ministry of health should coordinate and collaborate to train asthma educational experts so that patient motivation and activation can be enhanced during offline and online asthma educational programmes.
✓ It was found that traveling distances, internet accessibility issues, location and the number of asthma educational programmes are very low in rural areas of KSA. As a result, emergency visits had increased. Hospital management, SINA and the ministry of health must therefore invest in internet infrastructure as it can enhance the access to social media based asthma related content and resolve the issues of traveling distances, time and location management.
✓ Although patient motivation and activation has been analyzed at the pre-programme and post-programme stages, there is no mechanism to analyse the extent to which the study content itself plays role in enhancing patient activation and motivation during the undertaking of training programmes. Such a mechanism needs to be developed.
✓ The findings from the pre-program stage indicate that patients are not particularly aware about the benefits and the role of asthma educational programmes for enhancing their knowledge and skills. It was found that their social networks have lower levels of information about the purposes of asthma educational programmes. Therefore, SINA, hospital management, and the ministry of health must involve social actors, celebrities and experienced trainers to educate the rural areas community, especially where the challenges are high for asthma patients.
✓ There is no specific tool applied to evaluate the intrinsic and extrinsic motivation of program experts and patients during the asthma educational programme. Without analyzing the intrinsic and extrinsic motivation of both trainer and trainee, it is not possible to understand the challenges and maximize the benefits of asthma education in the context of Arab culture.
✓ Some patients have suggested that the motivation, commitment and experience of asthma educational programme experts and staff were not appropriate because they did not address the specific needs of patients through interactive discussions. Therefore, future asthma educational programmes must be designed so as to have maximum time duration and to address patient queries individually and comprehensively.
✓ Asthma educational programmes must be designed after considering the cultural and social aspects as well as the local context including the values and beliefs of patients. These aspects can enhance the acceptability, motivation, activation, and benefits of asthma education.

### Useful Influence in Public Policy

This study has highlighted the implications of asthma education with respect to patient activation, hospital staff training, content development and evaluation, patient motivation, patient knowledge, patient control, and patient self-management. This study will therefore be helpful for policy makers to address how patient activation and patient behavior can be improved through an asthma education program in MoH. This research is also helpful for understanding major issues in different contexts. These issues include the hospital setting, the environment, and the professional and patient contexts. Training is required for professionals to drive success. Therefore, the following major strategic recommendations should be noted by policy makers in the field of public health care.

**Figure 4:**
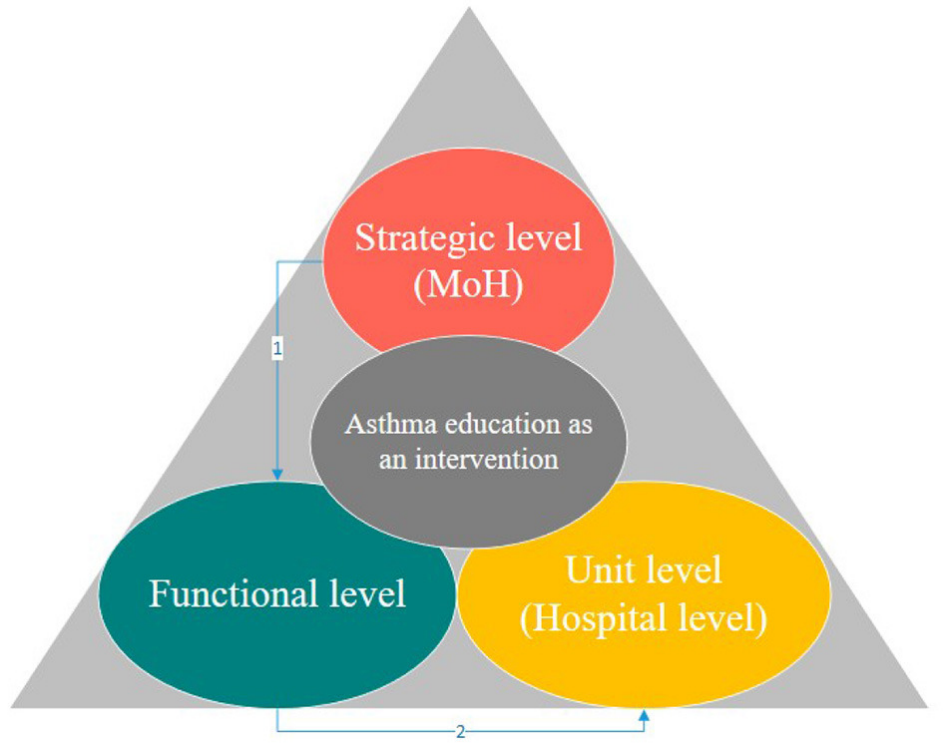
Policies level for implementation of asthma education as an intervention in Saudi Arabia (Developed by the author).

As shown in Figure 4, it would be useful to assign specific budgets to introduce an education program for asthma patients in Saudi Arabia. This requires top level (MoH) strategic level polices that can be implemented across the whole public health sector. Moreover, there is also a need for functional level polices like health education to offer patient education programmes to train professionals in the delivery of asthma education. Since, it was found that training staff in the design and delivery of asthma educational programmes, strategies are needed to engage patients, and to increase intrinsic and extrinsic motivation during asthma programmes. Hospital staff must be trained in asthma education delivery for patients.

There is a lack of research support available with respect to developing individual asthma education programmes in the KSA. Work is needed here in terms of content, educational program expertise, and staff training at the functional level of the MoH. The involvement of the researcher has been helpful to explore the challenges, as well as to use their extensive experience to obtain relevant data to meet the proposed objectives of this study. It was also found that hospital leaders and managers are not motivated to arrange such educational programmes. Therefore, at the functional level, the MoH should also seek to support such educational programmes. The researcher university (University of Salford) has provided some professional certificates for the professionals who participate in the programme and these have been found to motivate staff. Building on this, the HRM department of the MoD should offer policies to support learning and to encourage staff to participate in such programmes.

### Limitations and Future Direction

Every study offers useful practical recommendations as well as limitations. These limitations provide guidance to future researchers regarding how they can contribute to practice, theory, and society. It is also important that limitations should be highlighted because they can inversely influence the scope/generalizability or findings of any study.

There is no instrument available or designed which can evaluate patient activation and motivation during an asthma educational programme. These are some of the main limitations which may directly or indirectly influence the results of this study. Future researcher can adopt positivist epistemological and realist ontological positions to test the role of professional education, qualifications and hospital settings as regards patient activation during an asthma education pro programme. It is unclear whether the lower scores for women are a result of different trainers or other factors. As a result of the limitations of local culture, the researcher was not able to observe female professionals and patients. One of the more important limitations of this study is that the results cannot be generalized to lower social economic groups (i.e., those with less than high school/secondary education). However, the literacy rate of Saudi Arabia is above 95%, therefore there is small group of people who have less than high school/secondary education.

The present study was conducted after considering only one data collection method (i.e., survey method). Therefore, future researchers can explore the linkages between asthma educational programmes and patient self-management using mixed method approaches. Quantitative methods usually have a narrow focus and limit rich insights. Therefore, mixed methods are more appropriate for obtaining rich insights and analyzing the validity of a conceptual model (52, 53).

## Data Availability Statement

The datasets presented in this article are not readily available because the data is confidential and it cannot share it with anyone. Requests to access the datasets should be directed to drhamaddilah@gmail.com.

## Ethics Statement

The studies involving human participants were reviewed and approved by Saudi Ministry of Health (MOH). The patients/participants provided their written informed consent to participate in this study.

## Author Contributions

The author confirms being the sole contributor of this work and has approved it for publication.

## Conflict of Interest

The author declares that the research was conducted in the absence of any commercial or financial relationships that could be construed as a potential conflict of interest.

## Publisher's Note

All claims expressed in this article are solely those of the authors and do not necessarily represent those of their affiliated organizations, or those of the publisher, the editors and the reviewers. Any product that may be evaluated in this article, or claim that may be made by its manufacturer, is not guaranteed or endorsed by the publisher.

## Abbreviations

SINA: Saudi initiative for asthma
KAS: Kingdom of Saudi Arabia
WHO: World Health Organization
MOH: Saudi Ministry of Health.

## References

[1] World health Organization (WHO). Key Facts About Asthma. (2016). Available online at: https://www.who.int/news-room/fact-sheets/detail/asthma (accessed October 26, 2020).

[2] Pinnock H. Supported self-management for asthma. Breathe. (2015). 11:98–109. 10.1183/20734735.015614 26306110 PMC4487370

[3] Basyouni MH BinDhim NF Saini B Williams KA. Online health information needs for patients with asthma in Saudi Arabia. J Consum Health Internet. (2015). 19:13–24. 10.1080/15398285.2014.982050

[4] Naeem M Dailah HG. Facilitators, challenges and usefulness of an asthma educational programme. Health Educ J. (2020) 80:611–22. 10.1177/0017896920981620

[5] Alzayer R Almansour HA Basheti I Chaar B Al Aloola N Saini B. Asthma patients in Saudi Arabia-preferences, health beliefs and experiences that shape asthma management. Ethn Health. (2020) 1–17. 10.1080/13557858.2020.1817868 32931314

[6] Moradi-Lakeh M El Bcheraoui C Daoud F Tuffaha M Kravitz H Al Saeedi M. Prevalence of asthma in Saudi adults: findings from a national household survey. BMC Pulmon Med. (2015) 15:77. 10.1186/s12890-015-0080-5 26216220 PMC4517561

[7] Al Ghobain MO Algazlan SS Oreibi TM. Asthma prevalence among adults in Saudi Arabia. Saudi Med J. (2018). 39:179. 10.15537/smj.2018.2.20974 29436567 PMC5885095

[8] Alsswey A Al-Samarraie H Bervell B. mHealth technology utilization in the Arab world: a systematic review of systems, usage, and challenges. Health Technol. (2021) 11:1–13. 10.1007/s12553-021-00549-3

[9] Pinnock H Parke HL Panagioti M Daines L Pearce G Epiphaniou E. Systematic meta-review of supported self-management for asthma: a healthcare perspective. BMC Med. (2017) 15:64. 10.1186/s12916-017-0823-7 28302126 PMC5356253

[10] Warsi A Wang PS LaValley MP Avorn J Solomon DH. Self-management education programs in chronic disease: a systematic review and methodological critique of the literature. Arch Intern Med. (2004) 164:1641–9. 10.1001/archinte.164.15.1641 15302634

[11] Alahmadi TS Banjari MA Alharbi AS. The prevalence of childhood asthma in Saudi Arabia. Int J Pediatr Adolesc Med. (2019). 6:74–7. 10.1016/j.ijpam.2019.02.004 31388551 PMC6676310

[12] Al-Ghamdi BR Koshak EA Omer FM Awadalla NJ Mahfouz AA Ageely HM. Immunological factors associated with adult asthma in the Aseer Region, Southwestern Saudi Arabia. Int J Environ Res Public Health. (2019) 16:2495. 10.3390/ijerph16142495 31336954 PMC6678431

[13] Al-Zahrani JM Ahmad A Al-Harbi A Khan AM Al-Bader B Baharoon S. Factors associated with poor asthma control in the outpatient clinic setting. Ann Thorac Med. (2015) 10:100–4. 10.4103/1817-1737.152450 25829960 PMC4375737

[14] Bano R. The prevalence of asthma and its related risk factors among the children in Hail Area, Kingdom of Saudi Arabia. EC Pulmon Respir Med. (2019). 8:210–6. 10.4103/2278-0521.171436

[15] Mohammed A. Asthma prevalence among adults in Saudi Arabia. Saudi Med J. (2018). 39:740. 10.15537/smj.2018.7.23170 29968901 PMC6146246

[16] Elbanna RMH Sileem AE Bahgat SM Ibrahem GA. Effect of bronchial asthma education program on asthma control among adults at Mansoura district. Egypt J Chest Dis Tuberculosis. (2017) 66:561–9. 10.1016/j.ejcdt.2017.03.001

[17] Al-Moamary MS Alhaider SA Alangari AA Al Ghobain MO Zeitouni MO Idrees MM. Al-Hajjaj MS. The Saudi Initiative for Asthma-2019 Update: guidelines for the diagnosis and management of asthma in adults and children. Ann Thorac Med. (2019) 14:3–48. 10.4103/atm.ATM_327_18 30745934 PMC6341863

[18] Nguyen VN Huynh TTH Chavannes NH. Knowledge on self-management and levels of asthma control among adult patients in Ho Chi Minh City, Vietnam. Int J Gen Med. (2018). 11:81. 10.2147/IJGM.S157050 29520161 PMC5833772

[19] Al-Durra M Torio MB Cafazzo JA. The use of behavior change theory in Internet-based asthma self-management interventions: a systematic review. J Med Internet Res. (2015). 17:e89. 10.2196/jmir.4110 25835564 PMC4400315

[20] Mosnaim G Safioti G Brown R DePietro M Szefler SJ Lang DM. Merchant RK. Digital Health Technology in Asthma: a comprehensive scoping review. J Allergy Clin Immunol Pract. (2021) 9:2377–98. 10.1016/j.jaip.2021.02.028 33652136

[21] Mondragon P Ducharme FM. Asthma education and specialized care after pediatric emergency department visits: real-life impact. Can J Respir Crit Care Sleep Med. (2021) 1–21. 10.1080/24745332.2021.1896959

[22] Davis J Fitzmaurice L. Providing individualized written asthma action plans during the pediatric emergency department visit. J Asthma. (2021) 58:819–24. 10.1080/02770903.2020.1731824 32066290

[23] Bozigar M Lawson AB Pearce JL King K Svendsen ER. A Bayesian spatio-temporal analysis of neighborhood pediatric asthma emergency department visit disparities. Health Place. (2020) 66:102426. 10.1016/j.healthplace.2020.102426 33011491 PMC8591955

[24] Byrwa-Hill BM Venkat A Presto AA Rager JR Gentile D Talbott E. Lagged association of ambient outdoor air pollutants with asthma-related emergency department visits within the Pittsburgh region. Int J Environ Res Public Health. (2020) 17:8619. 10.3390/ijerph17228619 33233547 PMC7699695

[25] Rangachari P Chen J Ahuja N Patel A Mehta R. Demographic and Risk factor differences between children with “one-time” and “repeat” visits to the Emergency Department for Asthma. Int J Environ Res Public Health. (2021) 18:486. 10.3390/ijerph18020486 33435304 PMC7827100

[26] Becker JH Feldman JM Arora A Busse PJ Wisnivesky JP Federman AD. Cognition, symptom perception, and medication non-adherence in older adults with asthma. J Asthma. (2020) 1–14. 10.1080/02770903.2020.1856867 33249956 PMC8180526

[27] Patel MR Song PX Sanders G Nelson B Kaltsas E Thomas LJ. A randomized clinical trial of a culturally responsive intervention for African American women with asthma. Ann Allergy Asthma Immunol. (2017) 118:212–9. 10.1016/j.anai.2016.11.016 28034579 PMC6020037

[28] Patel J Gimeno Ruiz de Porras D Mitchell LE Patel RR De Los Reyes J Delclos GL. Work-related asthma among certified nurse aides in Texas. Workplace Health Saf. (2020) 68:491–500. 10.1177/2165079920914322 32364022 PMC8851373

[29] Musharrafieh U Tamim H Houry R AlBuhairan F. A nationwide study of asthma correlates among adolescents in Saudi Arabia. Asthma Res Pract. (2020) 6:1–8. 10.1186/s40733-020-00056-8 32514367 PMC7262750

[30] Boulet LP Boulay MÈ Gauthier G Battisti L Chabot V Beauchesne MF. Benefits of an asthma education program provided at primary care sites on asthma outcomes. Respir Med. (2015) 109:991–1000. 10.1016/j.rmed.2015.05.004 26162708

[31] Gibeon D Liam G Heaney C Brightling R Niven HM. Dedicated severe asthma services improve health-care use and quality of life. CHEST J. (2015) 148:870–6. 10.1378/chest.14-3056 25789861

[32] Steurer-Stey C Storch M Benz S Hobi B Steffen-Bürgi B Steurer J. Motivational training improves self-efficacy but not short-term adherence with asthma self-management: a randomized controlled trial. Primary Health Care Res Dev. (2015). 16:32–41. 10.1017/S1463423613000480 24480615

[33] Federman AD Martynenko M O'Conor R Kannry J Karp A Lurio J. Rationale and design of a comparative effectiveness trial of home-and clinic-based self-management support coaching for older adults with asthma. Contemp Clin Trials. (2015) 44:103–11. 10.1016/j.cct.2015.07.018 26238181

[34] Mohamed Hussain S Ayesha Farhana S Mohammed Alnasser S. Time trends and regional variation in prevalence of asthma and associated factors in Saudi Arabia: a systematic review and meta-analysis. BioMed Res Int. (2018) 2018:8102527. 10.1155/2018/8102527 29951546 PMC5989288

[35] Al Ghamdi BR Mahfouz AA Abdel Moneim I Khan MY Daffallah AA. Altitude and bronchial asthma in south-western Saudi Arabia. East Mediterr Health J. (2008) 14:17–23. 18557448

[36] Holley S Morris R Knibb R Latter S Liossi C Mitchell F. Barriers and facilitators to asthma self-management in adolescents: a systematic review of qualitative and quantitative studies. Pediatr Pulmonol. (2017) 52:430–42. 10.1002/ppul.23556 27717193

[37] Armour C Bosnic-Anticevich S Brillant M Burton D Emmerton L Krass I. Pharmacy Asthma Care Program (PACP) improves outcomes for patients in the community. Thorax. (2007) 62:496–592. 10.1136/thx.2006.064709 17251316 PMC2117224

[38] Axelsson M Lötvall J. Recent educational interventions for improvement of asthma medication adherence. Asia Pacific Allergy. (2012) 2:67–75. 10.5415/apallergy.2012.2.1.67 22348209 PMC3269604

[39] Bodenheimer T Lorig K Holman H Grumbach K. Patient self-management of chronic disease in primary care. JAMA. (2002) 288:2469–75. 10.1001/jama.288.19.2469 12435261

[40] Riehm KE Kwakkenbos L Carrier ME Bartlett SJ Malcarne VL Mouthon L. Validation of the self-efficacy for managing chronic disease scale: a scleroderma patient-centered intervention network cohort study. Arthritis Care Res. (2016) 68:1195–200. 10.1002/acr.22807 26619042

[41] Chien CL Liu YF Liu WT Lu CC Wang PC Chiang LL. Impact of multidisciplinary self-management education program in patients with chronic obstructive pulmonary disease: self-efficacy, exercise tolerance, and quality of life. In: B109. HIGHLIGHTS AND ADVANCES IN PULMONARY REHABILITATION. American Thoracic Society (2016). A7863 p.

[42] Zhang M Xv G Luo C Meng D Ji Y. Qigong yi jinjing promotes pulmonary function, physical activity, quality of life and emotion regulation self-efficacy in patients with chronic obstructive pulmonary disease: a pilot study. J Altern Complem Med. (2016) 22:81–7. 10.1089/acm.2015.0224 27487437

[43] Emme C Mortensen EL Rydahl-Hansen S Østergaard B Svarre Jakobsen A Schou L Phanareth K. The impact of virtual admission on self-efficacy in patients with chronic obstructive pulmonary disease - a randomised clinical trial. J Clin Nurs. (2014) 23:3124–37. 10.1111/jocn.12553 24476457

[44] Kukla M Salyers MP Lysaker PH. Levels of patient activation among adults with schizophrenia: associations with hope, symptoms, medication adherence, recovery attitudes. J Nerv Ment Dis. (2013) 201:339–44. 10.1097/NMD.0b013e318288e253 23538980

[45] Hibbard JH Greene J. What the evidence shows about patient activation: better health outcomes and care experiences; fewer data on costs. Health Affairs. (2013) 32:207–14. 10.1377/hlthaff.2012.1061 23381511

[46] Alexander JA Hearld LR Mittler JN Harvey J. Patient-physician role relationships and patient activation among individuals with chronic illness. Health Serv Res. (2012) 47:1201–23. 10.1111/j.1475-6773.2011.01354.x 22098418 PMC3423181

[47] Thalib L Al-Taiar A. Dust storms and the risk of asthma admissions to hospitals in Kuwait. Sci Total Environ. (2012). 433:347–51. 10.1016/j.scitotenv.2012.06.082 22819885

[48] Lim AS Stewart K Abramson MJ Walker SP Smith CL George J. Multidisciplinary Approach to Management of Maternal Asthma (MAMMA): a randomized controlled trial. Chest J. (2014) 145:1046–54. 10.1378/chest.13-2276 24522786

[49] Janson SL McGrath KW Covington JK Cheng SC Boushey HA. Individualized asthma self-management improves medication adherence and markers of asthma control. J Allergy Clin Immunol. (2009) 123:840–6. 10.1016/j.jaci.2009.01.053 19348923 PMC2729175

[50] Gardner A Kaplan B Brown W Krier-Morrow D Rappaport S Marcus L. National standards for asthma self-management education. Ann Allergy Asthma Immunol. (2015) 114:178–86. 10.1016/j.anai.2014.12.014 25744903

[51] Aslam U Muqadas F Imran MK. Exploring the sources and role of knowledge sharing to overcome the challenges of organizational change implementation. Int J Organ Anal. (2018). 10.1108/IJOA-07-2017-1189

[52] Aslam U Muqadas F Imran MK Saboor A. Emerging organizational parameters and their roles in implementation of organizational change. Journal of Organizational Change Management. (2018). 31:1084–104. 10.1108/JOCM-08-2017-0300

[53] Nathan RA Sorkness CA Kosinski M Schatz M Li JT Marcus P. Development of the asthma control test: A survey for assessing asthma control. J Allergy Clin Immunol. (2004) 113:59–65. 10.1016/j.jaci.2003.09.008 14713908

[54] Andrews KL Jones SC Mullan J. Asthma self management in adults: a review of current literature. Collegian. (2014) 21:33–41. 10.1016/j.colegn.2012.12.005 24772988

[55] Al-Motlaq M Sellick K. Development and validation of an asthma knowledge test for children 8-10 years of age. Child Care Health Dev. (2011) 37:123–8. 10.1111/j.1365-2214.2010.01133.x 20637018

[56] Lababidi H Hijaoui A Zarzour M. Validation of the Arabic version of the asthma control test. Ann Thorac Med. (2008) 3:44–7. 10.4103/1817-1737.39635 19561904 PMC2700459

[57] Chang T Zhang Y Shan Y Liu S Song X Li Z. A study on the information-motivation-behavioural skills model among chinese adults with peritoneal dialysis. J Clin Nurs. (2018) 27:1884–90. 10.1111/jocn.14304 29421850

[58] Grischott T Senn O Rosemann T Frei A Cornuz J Martin-Diener E. Efficacy of motivating short interventions for smokers in primary care (COSMOS trial): study protocol for a cluster-RCT. Trials. (2019) 20:81. 10.1186/s13063-018-3071-z 30683155 PMC6347802

[59] Alzahrani Emad Ali M Alqarni SA Almakrami Ismail Hassan H. Assessment of knowledge and adherence of pediatric residents to saudi initiative asthma (SINA) guidelines in saudi arabia. Egypt J Hosp Med. (2018) 70:686–91. 10.12816/0043825

[60] Saudi initiative for Asthma. Guidelines for the Diagnosis and Management of Asthma in Adults and Children. (2016). Available online at: http://sinagroup.org/download/SINA_Guidelines_2016.pdf (accessed October 07, 2017).

[61] Al-Moamary MS Al-Hajjaj MS Idrees MM Zeitouni MO Alanezi MO Al-Jahdali HH. The saudi initiative for asthma. Ann Thorac Med. (2009). 4:216–32. 10.4103/1817-1737.56001 19881170 PMC2801049

[62] Chen SY Sheu S Chang CS Wang TH Huang MS. The effects of the self-efficacy method on adult asthmatic patient self-care behavior. J Nurs Res. (2010) 18:266–74. 10.1097/NRJ.0b013e3181fbe33f 21139446

[63] Poureslami I Nimmon L Doyle-Waters M Rootman I Schulzer M Kuramoto L. Effectiveness of educational interventions on asthma self-management in Punjabi and Chinese asthma patients: a randomized controlled trial. J Asthma. (2012). 49:542–51. 10.3109/02770903.2012.682125 22715910

[64] Tousman S Zeitz H Taylor LD Bristol C. Development, implementation and evaluation of a new adult asthma self-management program. J Commun Health Nurs. (2007) 24:237–51. 10.1080/07370010701645893 18092916

[65] Jain N Satish K Abhyankar N Velayudhan N Gurunathan J. Repeated exacerbation of asthma: an intrinsic phenotype of uncontrolled asthma. Lung India. (2019) 36:131. 10.4103/lungindia.lungindia_434_17 30829247 PMC6410599

[66] Boyd A Yang CT Estell K Tuggle C Gerald LB Dransfield M. Feasibility of exercising adults with asthma: a randomized pilot study. Allergy Asthma Clin Immunol. (2012) 8:13. 10.1186/1710-1492-8-13 22863207 PMC3511803

[67] Cortes T. Using focus groups to identify asthma care and education issues for elderly urban-dwelling minority individuals. Appl Nurs Res. (2004) 17:207–12. 10.1016/j.apnr.2004.06.002 15343555

[68] Murray B O'Neill M. Supporting self-management of asthma through patient education. Br J Nurs. (2018) 27:396–401. 10.12968/bjon.2018.27.7.396 29634337

[69] Busse PJ McDonald VM Wisnivesky JP Gibson PG. Asthma across the ages: adults. J Allergy Clin Immunol Pract. (2020) 8:1828–38. 10.1016/j.jaip.2020.03.044 32499032

[70] Scherzer R Grayson MH. Heterogeneity and the origins of asthma. Ann Allergy Asthma Immunol. (2018) 121:400–5. 10.1016/j.anai.2018.06.009 29928982 PMC6237278

[71] Rush KL Hatt L Janke R Burton L Ferrier M Tetrault M. The efficacy of telehealth delivered educational approaches for patients with chronic diseases: a systematic review. Patient Educ Counsel. (2018) 101:1310–21. 10.1016/j.pec.2018.02.006 29486994

[72] Martynenko M O'Conor RM Wisnivesky JP Wolf M Federman A Mindlis I. Barriers to asthma self-management: results from the SAMBA screening instrument. In: C38. New Developments in Asthma Care, Methods and Outcomes. American Thoracic Society (2016). A4956 p.

[73] Hanlon P Daines L Campbell C McKinstry B Weller D Pinnock H. Telehealth interventions to support self-management of long-term conditions: a systematic metareview of diabetes, heart failure, asthma, chronic obstructive pulmonary disease, and cancer. J Med Internet Res. (2017) 19:e172. 10.2196/jmir.6688 28526671 PMC5451641

[74] Hoch HE Houin PR Stillwell PC. Asthma in children: a brief review for primary care providers. Pediatr Ann. (2019) 48:e103–9. 30874817 10.3928/19382359-20190219-01

[75] Weinstein SF Katial RK Bardin P Korn S McDonald M Garin M. Effects of reslizumab on asthma outcomes in a subgroup of eosinophilic asthma patients with self-reported chronic rhinosinusitis with nasal polyps. J Allergy Clin Immunol: Pract. (2019) 7:589–96. 30193936 10.1016/j.jaip.2018.08.021

